# Different responses of two maize cultivars to *Spodoptera frugiperda* (Lepidoptera: Noctuidae) larvae infestation provide insights into their differences in resistance

**DOI:** 10.3389/fpls.2023.1065891

**Published:** 2023-02-10

**Authors:** Jinwen Yang, Changlu Ma, Ru Jia, Haiyan Zhang, Yanming Zhao, Haiwang Yue, Heqin Li, Xuwen Jiang

**Affiliations:** ^1^ College of Agronomy, Qingdao Agricultural University, Qingdao, Shandong, China; ^2^ Dryland Farming Institute, Hebei Academy of Agriculture and Forestry Sciences, Hengshui, China; ^3^ Department of Biological Sciences, Royal Holloway University of London, Egham, United Kingdom

**Keywords:** *Spodoptera frugiperda*, plant defense, antioxidative enzyme, secondary metabolism, phytohormones

## Abstract

*Spodoptera frugiperda* (Lepidoptera: Noctuidae), a pest with an amazing appetite, damages many crops and causes great losses, especially maize. Understanding the differences in different maize cultivars’ responses to *S. frugiperda* infestation is very important for revealing the mechanisms involved in the resistance of maize plants to *S. frugiperda*. In this study, a comparative analysis of two maize cultivars, the common cultivar ‘ZD958’ and the sweet cultivar ‘JG218’, was used to investigate their physico-biochemical responses to *S. frugiperda* infestation by a pot experiment. The results showed that the enzymatic and non-enzymatic defense responses of maize seedlings were rapidly induced by *S. frugiperda*. Frist, the hydrogen peroxide (H_2_O_2_) and malondialdehyde (MDA) contents of infested maize leaves were significantly increased and then decreased to the level of the control. Furthermore, compared with the control leaves, the puncture force values and the total phenolics, total flavonoids, and 2,4-dihydroxy-7-methoxy-1,4-benzoxazin-3-one contents of infested leaves were significantly increased within a certain time. The superoxide dismutase and peroxidase activities of infested leaves were significantly increased in a certain period of time, while the catalase activities decreased significantly and then increased to the control level. The jasmonic acid (JA) levels of infested leaves were significantly improved, whereas the salicylic acid and abscisic acid levels changed less. Signaling genes associated with phytohormones and defensive substances including *PAL4*, *CHS6*, *BX12*, *LOX1*, and *NCED9* were significantly induced at certain time points, especially *LOX1*. Most of these parameters changed greater in JG218 than in ZD958. Moreover, the larvae bioassay showed that *S. frugiperda* larvae weighed more on JG218 leaves than those on ZD958 leaves. These results suggested that JG218 was more susceptible to *S. frugiperda* than ZD958. Our findings will make it easier to develop strategies for controlling *S. frugiperda* for sustainable maize production and breeding of new maize cultivars with increased resistance to herbivores.

## 1 Introduction

An herbivore attack causes serious destruction to plant growth and considerable loss in crop yield. Over the course of coevolution and interactions between plants and herbivores, host plants have developed a variety of defenses against herbivores (War et al., 2012). Host plant resistance is considered to be one of the important ways for reducing the losses, which can be constitutive or inducible (Fürstenberg-Hägg et al., 2013). In the absence of herbivores, constitutive defenses for herbivores are the fundamental expression of physical and chemical defensive traits, whereas induced defenses are only activated after an attack by herbivores (Anstett et al., 2016). Plant-induced defenses are very effective in defensing against following herbivore attacks (Mertens et al., 2021), which can be direct or indirect. Direct induced resistance is mediated by the accumulation of defense-related proteins to defend plants against herbivores, or plants may produce noxious chemicals to prevent herbivores from feeding, ovipositing, or growing (War et al., 2012); whereas indirect induced resistance causes the release of a mixture of volatile compounds that specifically attract the plant’s natural enemies (predators or parasitoids) (Aljbory and Chen, 2018).

Secondary metabolites, such as compounds containing nitrogen and sulfur, terpenoids, and phenolics, plant defensive proteins, which are connected in the activation and enhancement of defense systems in plants, are inducible defenses against herbivores (War et al., 2012; Divekar et al., 2022). In order to shield plants from herbivores, they are synthesized to perform defense functions and control defense signaling pathways. Many of these signal transduction pathways are mediated by a network of phytohormones, including jasmonic acid (JA), salicylic acid (SA), and abscisic acid (ABA) (Guo et al., 2019). The regulation of plant-induced resistance to herbivores, especially, is significantly influenced by the accumulation of JA and SA (Pieterse et al., 2012; Yang et al., 2015; Guo et al., 2019). These phytohormones may act individually, synergistically, or antagonistically to activate signal transduction upon herbivore feeding. Many genes are involved in these processes and metabolic pathways, such as *phenylalanine ammonia*-*lyase* (*PAL*) genes (Cass et al., 2015), *lipoxygenase* (*LOX*) genes (Njaci et al., 2020), *9*-*cis*-*epoxycarotenoid dioxygenase* (*NCED*) genes (Han et al., 2004), *benzoxazinoid* (*BX*) genes (Tzin et al., 2017), and *chalcone synthase* (*CHS*) genes (Dehghan et al., 2014). Knowing how the plant’s defensive traits are controlled and expressed by genes will be helpful in developing new breeds with enhanced defense properties against herbivores.

*Spodoptera frugiperda* (*S. frugiperda*), a species of agricultural pest known as the fall armyworm (Lepidoptera: Noctuidae), is widespread and disruptive. With their chewing mouthparts, *S. frugiperda* larvae consume crop seedlings, leaves, and kernels, thus reducing crop yields (Zhang et al., 2022). A wide range of crops are targeted by this pest, but several crops, including maize, rice, sorghum, turf grasses, cotton, and peanuts, are preferred (Carroll et al., 2006). One of the world’s most important agricultural crops, maize is frequently used as food, feed, and industrial raw material. Therefore, maize plays a crucial role in ensuring economic and food security. *S. frugiperda* has rapidly spread since it entered China in January 2019, infecting grain-producing regions in southern and southwestern China and posing a significant threat to the maize crop (Jing et al., 2020; Gao et al., 2021). It is urgent to make safe and effective control strategies for *S. frugiperda*.

One important method for controlling pests in agriculture is plant-induced resistance. In the past couple of decades, plant-induced resistance to various stresses has made significant progress and has emerged as an important topic in plant–herbivore interactions (War et al., 2011; War et al., 2012). Therefore, to study the maize defense response to *S. frugiperda* is very valuable for the utilization of host plant resistance to control *S. frugiperda*. To date, research on the response of host plants to *S. frugiperda* were focused on plant volatile compound identification, genetic resistance, host plant preference, *etc*. (Costa et al., 2020; De Lange et al., 2020; Garlet et al., 2021; Hafeez et al., 2021; Haq et al., 2021; Jacques et al., 2021; Lv et al., 2021; Murad et al., 2021; Yactayo-Chang et al., 2021; Peterson et al., 2022). However, the performance and the mechanism of maize plants in response to *S. frugiperda* still need to be studied further. Understanding the differences in different maize cultivars’ responses to *S. frugiperda* is vital to reveal the mechanisms involved in the resistance of maize plants to *S. frugiperda*. Therefore, the present study was performed using two different kinds of maize cultivars with different genetic backgrounds to assess the resistance differences to *S. frugiperda* feeding. The study includes larval weight gain, change of puncture force, accumulation of total phenolic compounds, total flavonoids, and 2,4-dihydroxy-7-methoxy-1,4-benzoxazin-3-one (DIMBOA), contents of hydrogen peroxide (H_2_O_2_) and malondialdehyde (MDA), activities of antioxidant enzymes superoxide dismutase (SOD), peroxidase (POD), and catalase (CAT), and levels of phytohormones jasmonic acid (JA), salicylic acid (SA), and abscisic acid (ABA) in the *S. frugiperda*-infested leaves and control (CK). The level of defense substances and phytohormone biosynthesis genes in response to *S. frugiperda* infestation was also assessed using gene expression analysis. It can provide a theoretical basis for developing crop cultivars and pest management for sustainable crop production.

## 2 Materials and methods

### 2.1 Plant materials

Two different kinds of maize cultivars with different genetic backgrounds were used. ‘Zhengdan958’ (ZD958) is one of the most common cultivars, which is the hybrid of Zheng58 × Chang7-2 and one of the most widely planted hybrid in China. ‘Jinguan218’ (JG218) is another sweet corn, which is the hybrid of Sweet62 × Sweet 601 and one of the most widely planted hybrid in China. The uncoated seeds of ZD958 and JG218, produced in the year 2021, were bought from Beijing Denong Seed Industry Co., Ltd. and Beijing Sihai Seed Industry Co., Ltd., respectively. The seeds were stored in a cool chamber at 10°C and relative humidity of 40%. All experiments were conducted using healthy 15-day-old ZD958 (~30 cm in height) and JG218 (~25 cm in height) cultivar seedlings. The maize seeds were sown in plastic pots (7 cm in height and 6 cm in diameter) with commercial nutrient soil (Pindstrup, Denmark) and placed in a climate-controlled room (temperature of 25 ± 1°C, relative humidity of 60 ± 5%) under 16 -h light/8-h dark photoperiods and supplied with the same amount of water for each pot every 2 or 3 days. Three plants of each maize cultivar per pot were retained after the plant emergence. When *S. frugiperda* was introduced, the maize plants were transferred into ventilated cages (70 × 70 × 50 cm). The cages were maintained in a climate-controlled condition as described above.

### 2.2 Experimental treatments

**Figure 1:**
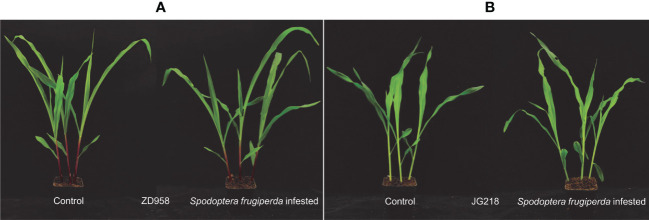
Infested and uninfested leaves (control) of maize from ZD958 **(A)** and JG218 **(B)**.

*S. frugiperda* larvae were reared from eggs provided by Henan Jiyuan Baiyun Industry Co., Ltd., Henan Province, China. The eggs were kept in an incubator (temperature of 27 ± 1°C, relative humidity of 30–40%) until the emergence of the neonate larvae. After that, they were moved to rear at room temperature (25 ± 1°C). Before feeding on maize plants, the third-instar larvae were starved for 1 h. Then, three larvae fed on the leaves of each plant at 2:00 PM. After 1 h, the larvae were removed to limit both the extent and the time frame of feeding damage (Carroll et al., 2006). The maize leaves were harvested at 0, 3, 6, and 12 h after the larvae were removed and then immediately used or stored at -80°C according to the object of the study. All biochemical parameters were determined on both the infected and uninfected (control) leaves of each cultivar (**Figure 1**). At each time point, three biological replicates of the experiment were set up, and plants of three pots were used for each replicate.

### 2.3 Larvae bioassay

After having been starved for 2 h, the third-instar larvae randomly selected were placed in 90-mm-diameter petri dishes with enough freshly detached leaves of ZD958 and JG218 for 24 h. A single third-instar larva was placed in each petri dish (Njaci et al., 2020). A total of 10 petri dishes were used as a biological replicate, with a total of 30 petri dishes for three biological replicates. All petri dishes were placed at room temperature (temperature of 25 ± 1°C, relative humidity of 60 ± 5%) with 16-h light/8-h dark photoperiod. The body weight of the larvae was determined using an automatic electro-balance every 3 h.

### 2.4 Measurement of puncture force (F) of maize leaves

A Handpi screen view electric test stand (Yueqing Handpi Instruments Co., Ltd., China) with a cylindrical probe of 0.5 mm in diameter and 5 mm in length was used to puncture the leaf samples. The measurement range of the instrument is set to 0–10 N. The leaf samples were set tightly on the stage with clips, and the probe moved down toward the stage at a speed of 30 mm/min. A creep meter that was outfitted with force analysis software for automatic computer analysis was used to measure the puncture force values (N). Higher values indicated that more force was required to puncture the tissues, while lower values indicated that crispness was higher. Three biological replicates were set up; for each replicate, measurements were taken separately from three pieces (~4 cm in length of the damaged leaf or the counterpart of the control leaf) of each of the middle region of the second and third leaf, respectively, and the average of 10 values was used to calculate the puncture force value of each segment. The sample’s puncture force value was calculated by taking the averages of the three pieces in each replicate. The above-mentioned procedure was carried out at room temperature (Gutiérrez-Rodríguez et al., 2013).

### 2.5 Measurement of H2O2 concentration of maize leaves

Based on a previous method (Quandahor et al., 2021), 0.15 g of leaf samples in an ice bath was homogenized with 5 ml of 0.1% (w/v) trichloroacetic acid (TCA). Then, after centrifugation at 10,000 × *g* for 10 min, the supernatant (0.5 ml) was mixed with 0.5 ml of 10 mM potassium phosphate (K_3_PO_4_) buffer (pH = 6.8) and 1 ml of 1 M potassium iodide. After 5 min, the supernatant’s absorption was read at a wavelength of 390 nm using a spectrophotometer (UV- Cary60, Agilent, USA). This step was repeated three times per biological replicate for a total of three biological replicates.

### 2.6 Determination of the MDA content of maize leaves

The MDA content was measured following a previously described method with slight modifications (Ahmad et al., 2021). Fresh leaf sample (0.5 g) was homogenized in 5.0 ml of 10% TCA solution and centrifuged at 4,000 × *g* for 10 min, and the supernatant was moved to new tubes. Then, 2 ml of the supernatant was mixed with 2 ml of 0.6% TCA solution. The mixture was thoroughly vortexed, heated for 15 min in boiling water, cooled immediately, and centrifuged for 5 min at 10,000 × *g*. Using a spectrophotometer (UV- Cary60, Agilent, USA), the absorbance values were measured at 532-, 450-, and 600-nm wavelengths. Three technical repeats were performed per biological replicate for a total of three biological replicates.

### 2.7 Extraction and determination of the total phenolic and flavonoid contents of maize leaves

The fresh leaf samples were ground to homogenize in liquid nitrogen. Then, 1.0 g each of the powder samples were extracted by ultrasound for 60 min at 40°C using 10 ml of 80% methanol, and the supernatant of the extracted samples were obtained after centrifugation at 7,000 × g for 20 min. Folin–Ciocalteu reagent was used to detect the total phenolic compound content. The samples (0.5 ml) were mixed with 2.5 ml of a 10-fold diluted Folin–Ciocalteu reagent and 2 ml of 7.5% sodium carbonate in tubes. Before being measured with a spectrophotometer (UV- Cary60, Agilent, USA) at 760 nm, the tubes were covered and left to stand for 30 min at room temperature (Złotek et al., 2014). Gallic acid (0 to 15 μg/ml) was used as standard solution to determine the total phenolic content. The total flavonoid contents of the leaf extracts were determined using aluminum chloride reagent. In brief, 1.5 ml of 80% methanol, 0.1 ml of 10% aluminum chloride hexahydrate, 0.1 ml of 1 M sodium acetate, and 2.8 ml of deionized water were combined with 0.5 ml of the extract solution. After 40 min, at a wavelength of 415 nm, the mixture absorption of the control was read (Złotek et al., 2014). Using the same method as described above, the standard curve for total flavonoids was established using the rutin standard solution ranging from 0 to 50 µg/ml. Three technical repeats were performed per biological replicate for a total of three biological replicates.

### 2.8 Determination of the benzoxazine content of maize leaves

A previously described method was used to measure the content of benzoxazine in maize leaves, with some slight modifications (Bao et al., 2021). In short, 2 ml of 70% acetonitrile and 1% acetic acid in water were used to oscillate 0.5 g of fresh maize leaves for 2 h at 60°C. After being centrifuged at 4,000 × *g* for 10 min at room temperature, the mixture solutions were filtered through 0.45-μm filters. Then, at 40°C, 10 μl of the filtrate was analyzed by liquid chromatography (Agilent 1100, USA) equipped with UV spectra (DAD detector) and a Xtimate C18 column (250 mm × 4.6 mm i.d., 5 μm; Yuexu, China). Furthermore, 280 nm was selected as the wavelength, and 0.01% phosphoric acid in water (phase A) and acetonitrile (phase B) made up the mobile phase. The following was set up for the gradient elution program: 0–0.01 min at 5% B, 0.01–32 min at 5–30% B, 32–36 min at 30–80% B, 36–37 min at 80–5% B, and 37–46 min at 5% B. Additionally, the DIMBOA content (in μg/g fresh weight) was calibrated using standards of 20 μg/ml DIMBOA (Shanghai Yuanye Bio-Technology Co., Ltd., China). One technical repeat was performed per biological replicate for a total of three biological replicates.

### 2.9 Determination of the antioxidant enzyme activity of maize leaves

Under liquid nitrogen, 0.5 g of fresh leaf sample was homogenized with a mortar and pestle in each treatment using 5 ml of 100 mM precooled phosphate buffer (pH 7.4) containing 1 mM ethylenediamine tetraacetic acid and 5% polyvinylpyrrolidone. At 4°C, the crude homogenate was centrifuged for 10 min at 5,000 × *g*. The supernatant was used to detect the SOD, POD, and CAT activities. The absorbance was read using a spectrophotometer (UV- Cary60, Agilent, USA). The ability of SOD to prevent the photochemical reduction of nitroblue tetrazolium (NBT) at 560 nm was used to determine its activity. A reduction of NBT of approximately 50% was considered to be one unit of enzyme activity (He et al., 2014). The optical density change per minute at 470 nm was used to calculate the POD activity (Zhang et al., 2020). The H_2_O_2_ decomposition was used to measure the CAT activity as a decline in the absorbance at 240 nm (He et al., 2014). The absorption values were repeated three times per biological replicate for a total of three biological replicates.

### 2.10 Plant hormone quantification of maize leaves

The plant hormones were extracted from 0.1 g of leaves using 0.9 ml of phosphate-buffered saline (pH 7.4). The crude homogenate was subjected to centrifugation at 3,000 × *g* for 15 min at 4°C. The supernatant was used to measure the contents of JA, SA, and ABA using commercially available assay kits (from Hefei Laier Biotechnology Co., Ltd., Anhui Province, China) according to the user manual provided by the manufacturer. The absorbance was read using a multifunctional ultraviolet fluorescent enzyme-labeled instrument (TECAN Infinite 200, Switzerland). Three technical repeats were performed per biological replicate for a total of three biological replicates.

### 2.11 Plant hormones and secondary metabolite-related gene expression analyses of maize leaves

Real-time quantitative PCR (qRT-PCR) was used to examine gene expression. Huayueyang Quick RNA Isolation Kit (Huayueyang Biotechnology, Beijing, China) was used to extract the total RNA of each sample in accordance with the protocol provided by the manufacturer. Reverse transcription was carried out using HiScript^®^ Q RT SuperMix for qPCR (+ gDNA wiper) (Vazyme, Nanjing, China). PCR was performed using ChamQ SYBR qPCR Master Mix (Vazyme) as described previously (Li et al., 2018). The gene-specific primers are listed in **Supplementary File S1**. *ZmActin* (*Zm00001d010159*) was used as an internal control. StepOnePlusTM Real-Time PCR system (Applied Biosystems, Ghent, Belgium) was used for qRT-PCRs, with three technical replicates for each biological replicate in a total of three biological replicates. The gene expression level in the infestation leaves at 0 h was set to 1, and the levels in other infested and control leaves were given relative to this for each cultivar. Using the 2^−ΔΔCt^ method (Livak and Schmittgen, 2001), the relative expression levels of the genes were determined.

### 2.12 Data analyses

All experimental data were presented as mean ± standard deviations (SD) from repeat assays. The data was analyzed statistically with one-way ANOVA using SPSS 11.0. The differences of these parameters between the *S. frugiperda*-infested and the control leaves were compared using the Tukey test. *P <*0.05 was considered significant for differences.

## 3 Results

### 3.1 Weight gain of *S. frugiperda* larvae

**Figure 2:**
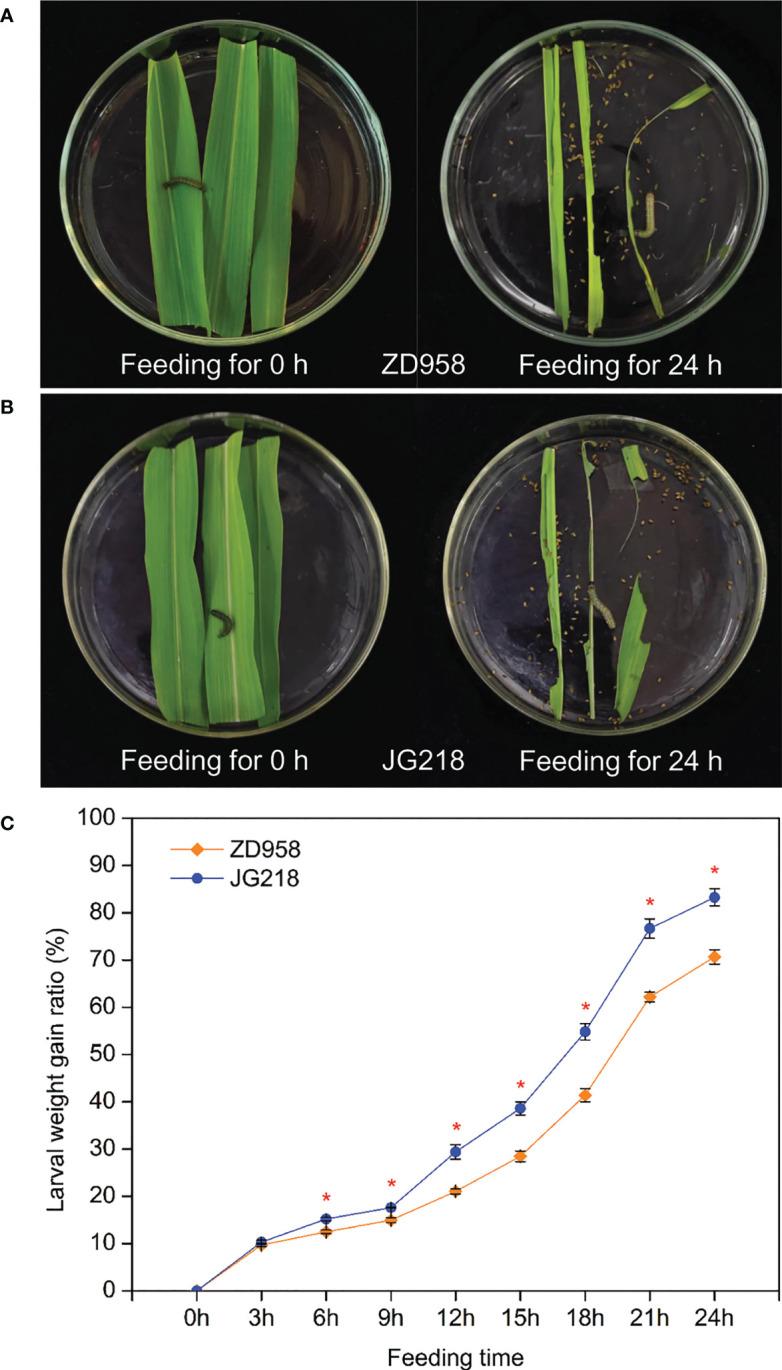
Larval weight gain for *S. frugiperda* fed on the detached leaves of ZD958 **(A)** and JG218 **(B)**. **(C)** Larval weight gain ratio. Data are shown as means ± SD (*n* = 3, every *n* represents the mean of 10 values). The asterisk indicates significant differences (*P* < 0.05) between ZD958 and JG218.

To investigate the response of different maize cultivars to *S. frugiperda* infestation, a larvae bioassay on the detached leaves of ZD958 and JG218 was carried out. In order to make the larvae much hungrier and discharge more feces or urine, they were starved and used after 2 h. The results showed that the differences between the detached leaves of ZD958 and JG218 on larval weight gain were significant from 6 h onwards after *S. frugiperda* was fed (*F* = 27704.287, *df* =17, *P* = 0.000). A higher larval weight gain ratio was observed on the JG218 leaves (0–83.28%) compared with those from the ZD958 leaves (0–70.64%) (**Figure 2**). It suggests that JG218 was more susceptible to *S. frugiperda* than ZD958. The different genetic backgrounds of ZD958 and JG218 may be the reason.

### 3.2 Mechanical properties of maize leaves

**Figure 3:**
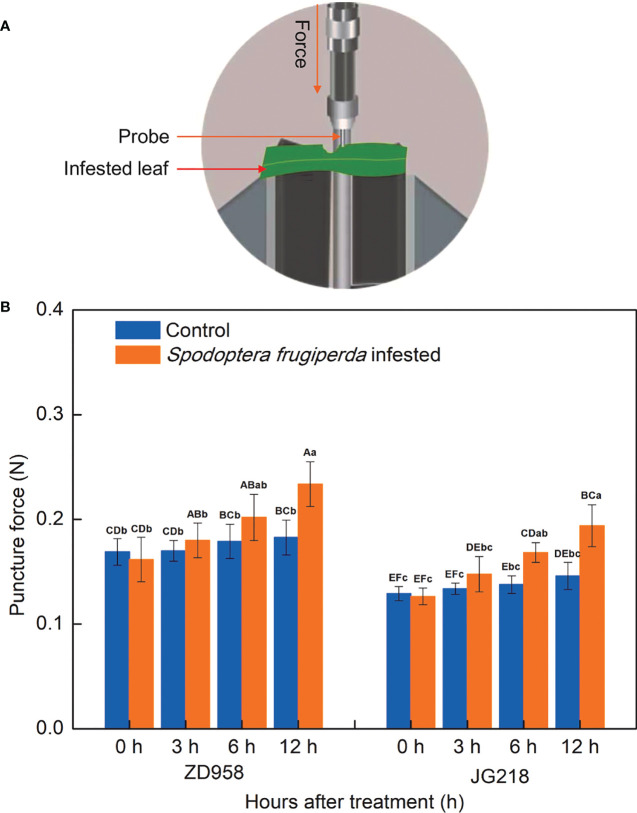
Effect of *S. frugiperda* infestation on puncture force levels in maize leaves. **(A)** Force needed to puncture the leaf. **(B)** Puncture force levels. Data are shown as means ± SD (*n* = 3, every *n* represents the mean of three pieces of each of the middle region of the second and third leaf, respectively). Different letters indicate significant differences at *P* < 0.05. Capital letters represent differences between the two maize cultivars, while lowercase letters indicate differences between treatments within the same cultivar.

The mechanical properties of maize leaves were analyzed using puncture force. The change trend of puncture force in both maize cultivars was quite similar. Notably, the level of puncture force increased in both maize cultivars from 3 h onwards after *S. frugiperda* infestation, relative to the control leaves, reached the maximum, and had a statistically significant difference at 12 h (**Figure 3**). The increase ranged from 5.88% to 27.92% in ZD958 (*F* = 2.74, *df* =7, *P* = 0.045) and from 10.47% to 32.88% in JG218 (*F* = 4.81, *df* =7, *P* = 0.004), respectively. In both the *S. frugiperda*-infested and the control leaves, the levels of puncture force in ZD958 were significantly higher than that in JG218 at the same point of time. It suggested that more force was required to puncture the tissues of ZD958 than that of JG218.

### 3.3 H_2_O_2_ and MDA contents in maize leaves

**Figure 4:**
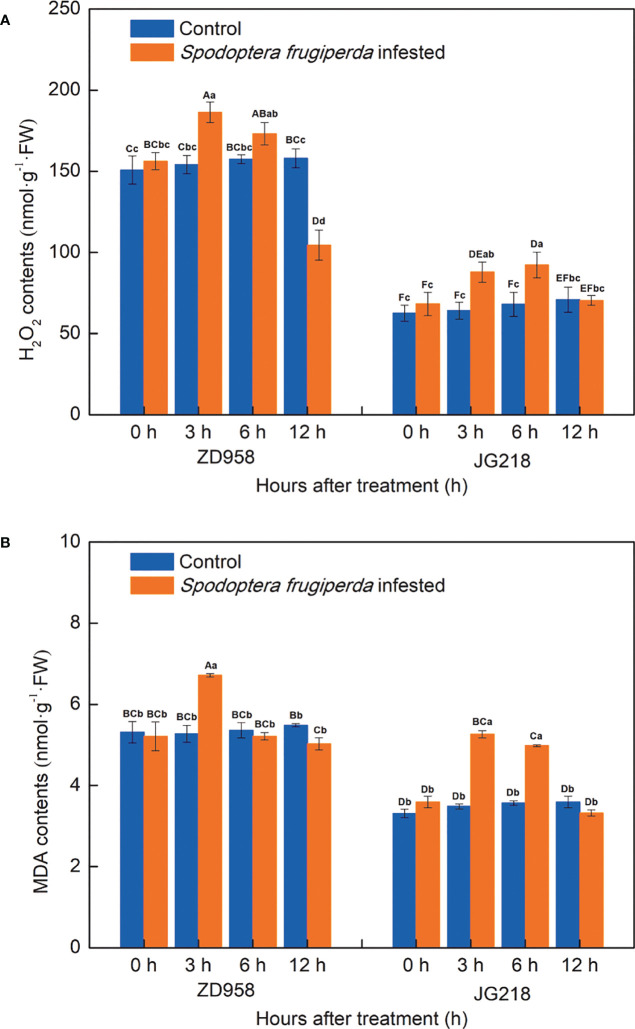
Effect of *S. frugiperda* infestation on H_2_O_2_ **(A)** and malondialdehyde **(B)** concentration in maize leaves. Data are shown as means ± SD (*n* = 3, every *n* represents the mean of three seedlings). Different letters indicate significant differences at *P* < 0.05. Capital letters represent differences between the two maize cultivars, while lowercase letters indicate differences between treatments within the same cultivar.

After *S. frugiperda* infestation, the H_2_O_2_ concentration in both maize cultivars tended to first increase and then decrease from 0 h onwards, with a little difference between the two cultivars (**Figure 4A**). Compared with the control leaves, the H_2_O_2_ concentration in the infested leaves of ZD958 first significantly increased the maximum at 3 h by the largest proportion to 21.00% and then significantly decreased the lowest at 12 h with the proportion to 33.88% (*F* = 14.191, *df* =7, *P* = 0.000). The H_2_O_2_ concentration in the infested leaves of JG218 was first significantly increased, reached a maximum at 6 h by the proportion to 35.77% (*F* = 2.932, *df* =7, *P* = 0.035), and then decreased to the level of the control at 12 h. Meanwhile, after *S. frugiperda* infestation, the MDA contents in both maize cultivars also tended to first increase and then decrease to the level of the control from 3 h onwards but remained lower compared with the control at 12 h (**Figure 4B**). Compared with the control leaves, the MDA content in the *S. frugiperda*-infested leaves was significantly higher at 3 h for ZD958 (*F* = 7.264, *df* =7, *P* = 0.001) while significantly higher at 3 and 6 h for JG218 (*F* = 63.849, *df* =7, *P* = 0.000). At 3 h after *S. frugiperda* infestation, the MDA contents in ZD958 and JG218 increased by the largest proportion to 27.32% and 51.09%, respectively.

### 3.4 Total phenolic and flavonoid contents in maize leaves

**Figure 5:**
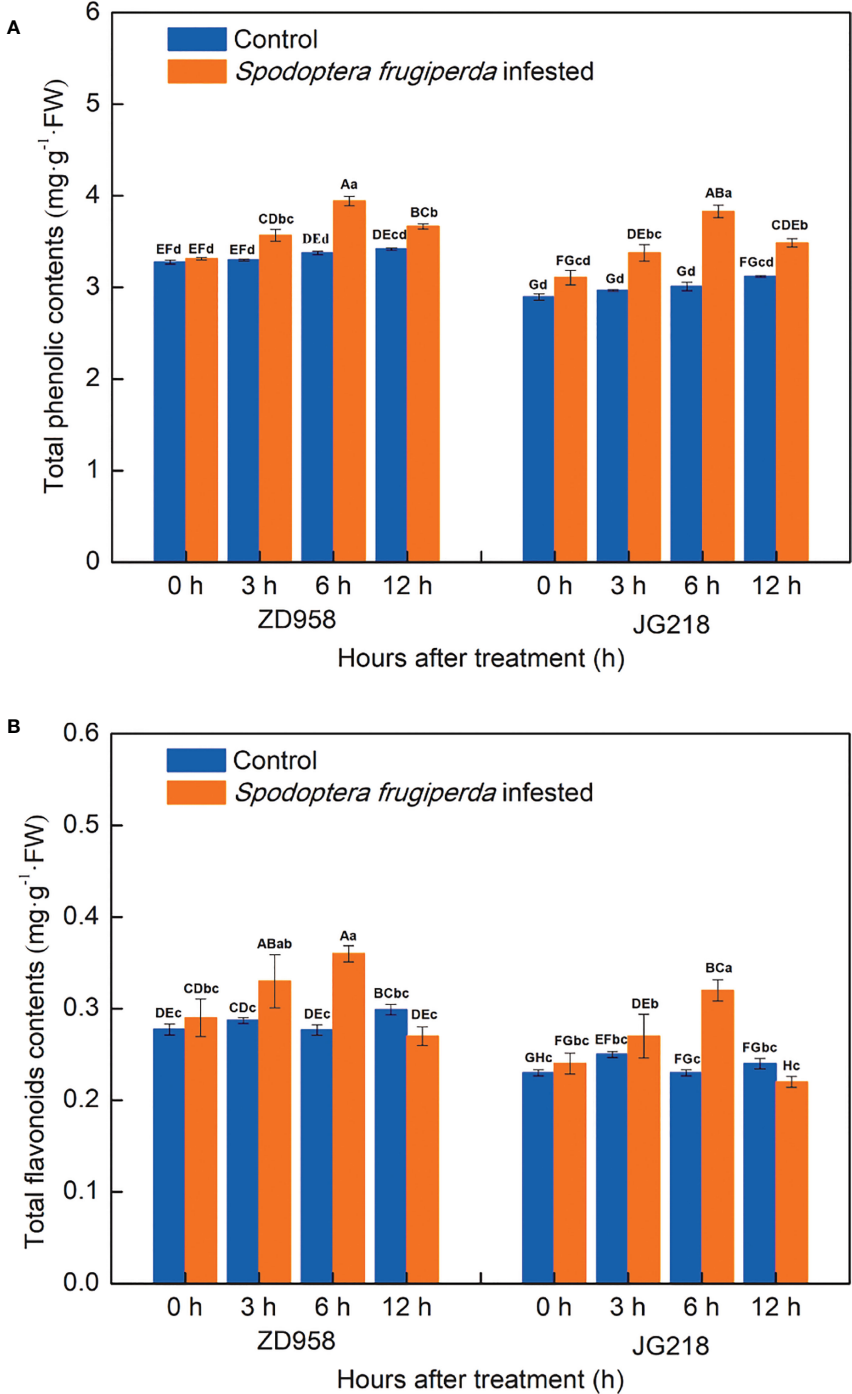
Effect of *S. frugiperda* infestation on total phenolic compound **(A)** and flavonoid **(B)** contents in maize leaves. Data are shown as means ± SD (*n* = 3, every *n* represents the mean of three seedlings). Different letters indicate significant differences at *P* < 0.05. Capital letters represent differences between the two maize cultivars, while lowercase letters indicate differences between treatments within the same cultivar.

A greater difference existed in the total phenolic contents of the *S. frugiperda*-infested leaves and the control leaves. In both maize cultivars, the total phenolic contents of the *S. frugiperda*-infested leaves tended to first increase and then decrease with prolonged time, reached a maximum at 6 h, but remained higher compared with the control at 12 h, and the differences were significant from 3 to 12 h (**Figure 5A**). The total phenolic contents in the *S. frugiperda*-infested leaves of ZD958 and JG218 increased from 1.12% to 16.78% (*F* = 48.731, *df* =7, *P* = 0.000) and from 7.25% to 27.24% (*F* = 31.741, *df* =7, *P* = 0.000), respectively. Similarly, the change trend of the total flavonoid contents was the same between the two maize cultivars. The total flavonoid contents of the *S. frugiperda*-infested leaves also tended to first increase and then decrease to the level of the control, reached a maximum at 6 h, and remained lower compared with the control at 12 h, but the differences were significant at 3 and 6 h for ZD958 and only at 6 h for JG218 (**Figure 5B**). The increase ranged from 15.40% to 30.34% in ZD958 (*F* = 5.185, *df* =7, *P* = 0.003) and from 8.28% to 37.15% in JG218 (*F* = 8.461, *df* =7, *P* = 0.000), respectively.

### 3.5 DIMBOA contents in maize leaves

**Figure 6:**
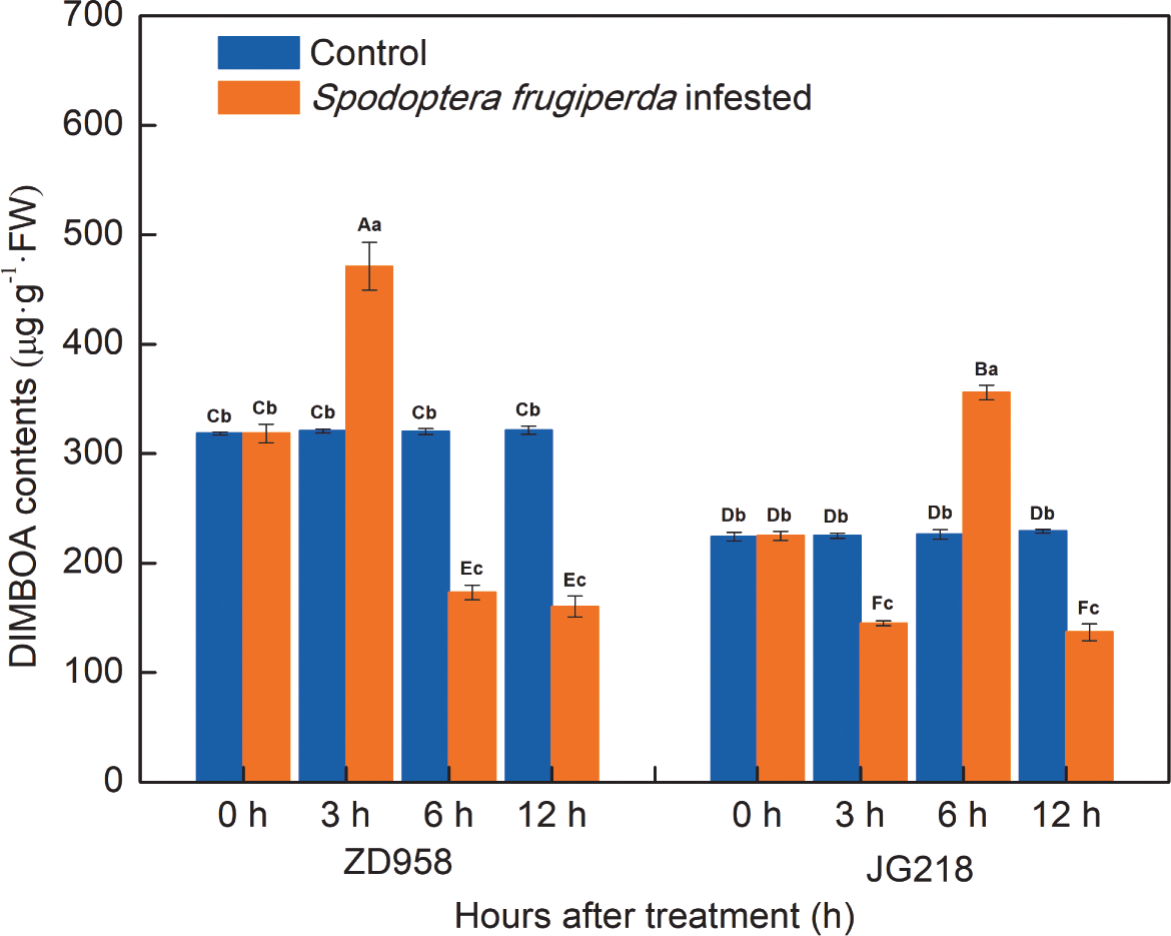
Effect of *S. frugiperda* infestation on 2,4-dihydroxy-7-methoxy-1,4-benzoxazin-3-one contents in maize leaves. Data are shown as means ± SD (*n* = 3, every *n* represents the mean of three seedlings). Different letters indicate significant differences at *P* < 0.05. Capital letters represent differences between the two maize cultivars, while lowercase letters indicate differences between treatments within the same cultivar.

The change trend of DIMBOA contents in both maize cultivars was different (**Figure 6**). Compared with the control, DIMBOA contents in the infested leaves of ZD958 first significantly increased and then significantly decreased, reached a maximum at 3 h by the largest proportion to 46.85%, and decreased the lowest value at 12 h by the proportion to 50.10% (F = 278.711, df =7, *P* = 0.000); while DIMBOA contents in the infested leaves of JG218 were first significantly decreased, then significantly increased, and significantly decreased at the late time, with a maximum at 6 h and the largest proportion to 57.27%, a minimum at 3 h with the proportion to 35.59% (F = 222.149, df =7, *P* = 0.000). Both the *S. frugiperda*-infested and the control leaves, from 0 h onwards, the DIMBOA contents in ZD958 were significantly higher than that in JG218 at the same point of time, except for at 6 h in the *S. frugiperda*-infested leaves.

### 3.6 Antioxidant enzyme activity in maize leaves

**Figure 7:**
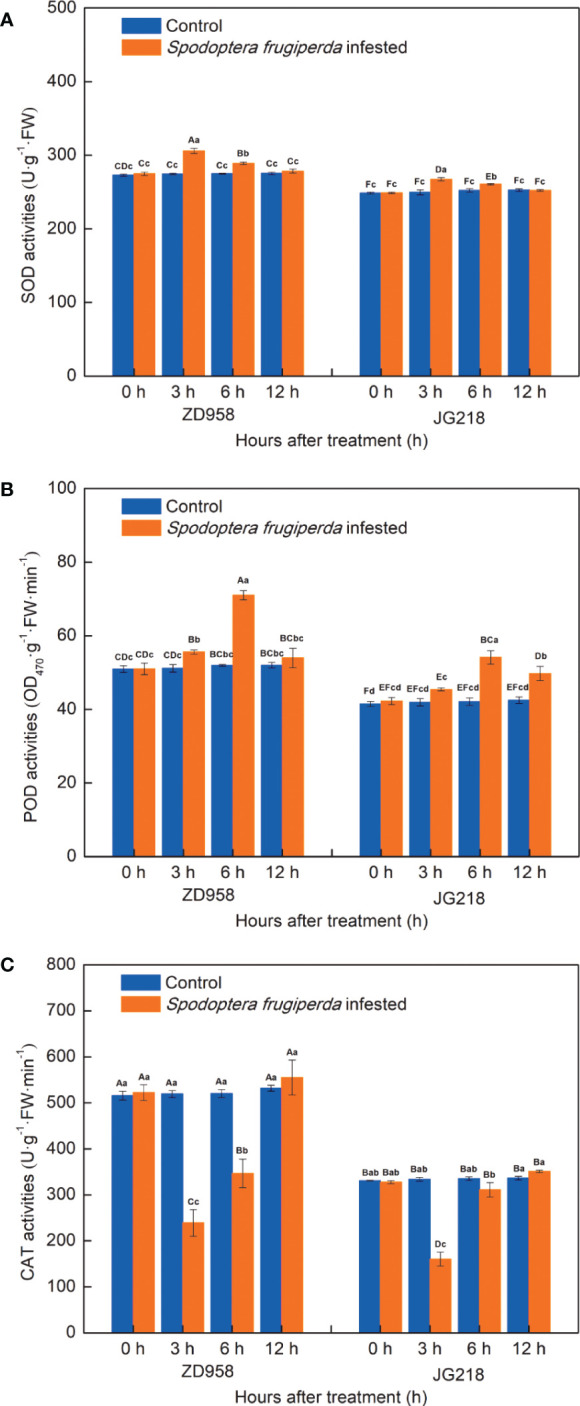
Effect of *S. frugiperda* infestation on antioxidant enzyme activity in maize leaves. **(A)** superoxide dismutase activity. **(B)** Peroxidase activity. **(C)** Catalase activity. Data are shown as means ± SD (*n* = 3, every *n* represents the mean of three seedlings). Different letters indicate significant differences at *P* < 0.05. Capital letters represent differences between the two maize cultivars, while lowercase letters indicate differences between treatments within the same cultivar.

After *S. frugiperda* infestation, the change trend of SOD activities in the leaves of both maize cultivars is similar, tended to first increase and then decrease to the level of the control, with the maximum at 3 h (**Figure 7A**). The SOD activities in the infested-leaves of both maize cultivars were significantly higher at 3 and 6 h compared with the control, but no statistically significant difference at 12 h. The increase ranged from 3.86% to 17.54% in ZD958 (F = 36.667, df =7, *P* = 0.001) and from 8.48% to 39.68% in JG218 (F = 53.234, df =7, *P* = 0.000), respectively. Similarly, POD activities in the infested-leaves of both maize cultivars also tended to first increase and then decrease, reached the maximum at 6 h, but a little differences between two cultivars (**Figure 7B**). Compared with the control, POD activities in the infested-leaves were significantly higher for ZD958 at 3 and 6 h (F = 10.111, df =7, *P* = 0.001), and higher but no significant difference at 12 h; while significantly higher for JG218 at 6 and 12 h (F = 49.806, df =7, *P* = 0.001). The increase ranged from 5.02% to 25.50% in ZD958 and from 6.68% to 32.74% in JG218, respectively. By contrast, the change trend of CAT activities in the leaves of both maize cultivars was different with the SOD and POD activities, tended to first significantly decrease (F = 27.066, df =7, *P* = 0.000 for ZD958; F = 56.514, df =7, *P* = 0.001 for JG218) and then increase to slightly higher than the control but no significant difference and showed a minimum at 3 h (**Figure 7C**). Both the *S. frugiperda*-infested and the control leaves, from 0 h onwards, the SOD, POD and CAT activities in ZD958 were significantly higher than that in JG218 at the same point of time, except for the CAT activity at 6 h in the *S. frugiperda*-infested leaves.

### 3.7 JA, SA, and ABA contents in maize leaves

**Figure 8:**
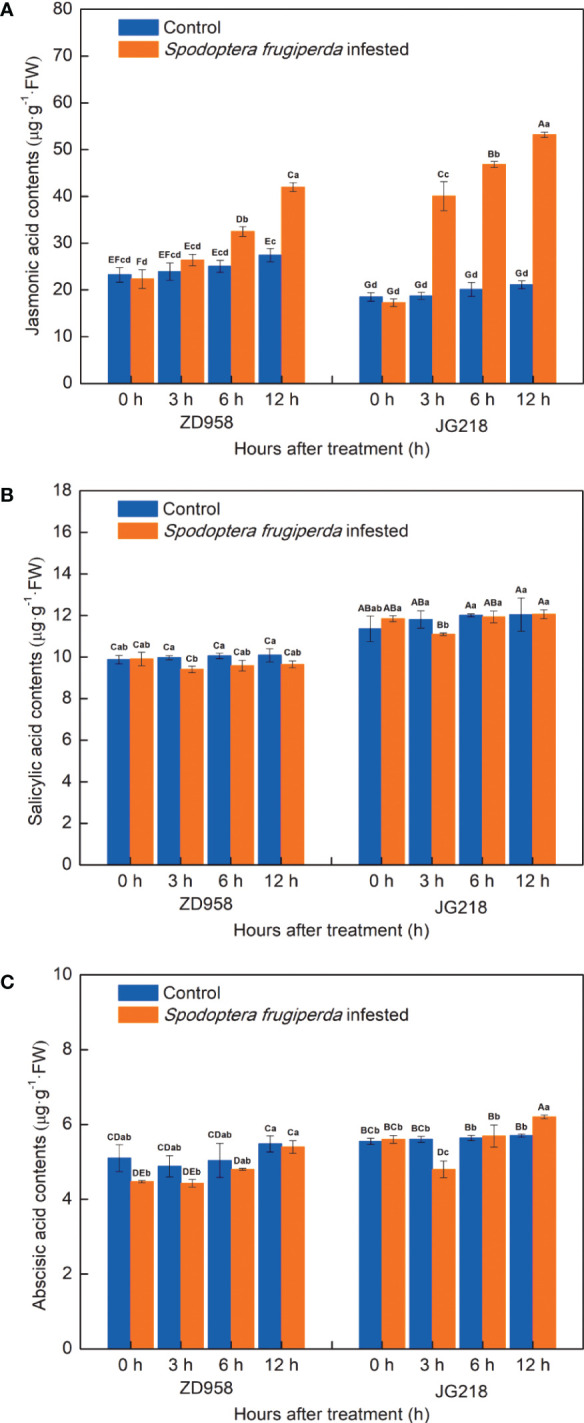
Effect of *S. frugiperda* infestation on salicylic acid **(A)**, jasmonic acid **(B)**, and abscisic acid **(C)** contents in maize leaves. Data are shown as means ± SD (*n* = 3, every *n* represents the mean of three seedlings). Different letters indicate significant differences at *P* < 0.05. Capital letters represent differences between the two maize cultivars, while lowercase letters indicate differences between treatments within the same cultivar.

In order to investigate the endogenous plant hormones in response to *S. frugiperda* infestation, we analyzed the contents of JA, SA and ABA. Notably, the level of endogenous JA significantly increased in JG218 (F = 113.967, df =7, *P* = 0.000) from 3 h onwards while in ZD958 (F = 20.055, df =7, *P* = 0.000) from 6 h onwards after *S. frugiperda* infestation, relative to the control leaves (**Figure 8A**). At 12 h after *S. frugiperda* infestation, the levels of JA in the *S. frugiperda*-infested leaves of ZD958 and JG218 were 1.53 and 2.52 times as high as the control, respectively. By contrast, the levels of SA in both maize cultivars do not appear to be different between the *S. frugiperda*-infested and the control leaves, except for a significant decrease at 3 h (F = 1.956, df =7, *P* = 0.126 for ZD958; F = 0.737, df =7, *P* = 0.645 for JG218) (**Figure 8B**). Similarly, compared with the control, the levels of ABA in the infested leaves of both maize cultivars were first decreased and then increased, and remained lower at the late time in ZD958 (F = 2.382, df =7, *P* = 0.071), but significantly lower at 3 h and significantly higher at 12 h in JG218 (F = 6.836, df =7, *P* = 0.001) (**Figure 8C**). It indicated that JA might play a central role in response to *S. frugiperda* infestation and ABA might be involved.

### 3.8 Phytohormones and secondary metabolite-related gene expression

**Figure 9:**
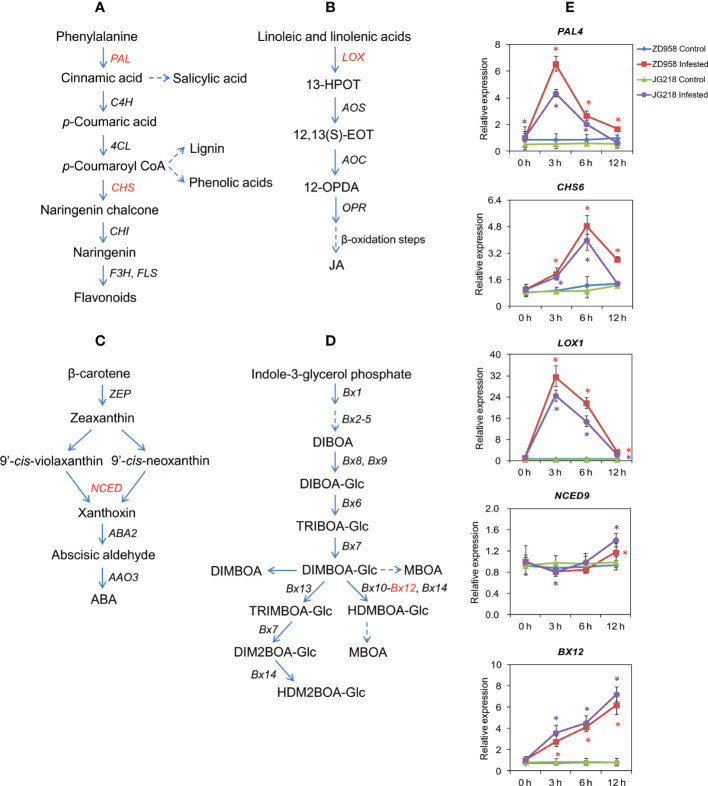
Effect of *S. frugiperda* infestation on phytohormones and secondary metabolite-related gene expression in maize leaves. **(A)** Biosynthetic pathway of flavonoids and salicylates. **(B)** Biosynthetic pathway of jasmonic acid. **(C)** Biosynthetic pathway of abscisic acid. **(D)** Biosynthetic pathway of benzoxazinoid. **(E)** qRT-PCR analysis of phytohormones and secondary metabolite-related genes. Data are shown as means ± SD (*n* = 3, every *n* represents the mean of three seedlings). Significant differences at *P* < 0.05 between the control and *S. frugiperda*-infested groups are indicated with an asterisk. The red asterisk represents significant differences for ZD958 and the purple asterisk for JG218.

PAL and CHS, the key enzymes in phenylpropanoid pathway, are involved in the biosynthesis of phenolics, flavonoids and SA (**Figure 9A**). After *S. frugiperda* infestation, the expression level of *PAL4* (*Zm00001d051166*) gene had a significant difference between the *S. frugiperda*-infested and the control leaves from 3 h onwards for ZD958 (F = 413.498, df =7, *P* = 0.000), while a significant difference between the *S. frugiperda*-infested and the control leaves from 0 to 6 h for JG218 (F = 1041.224, df =7, *P* = 0.000) (**Figure 9E**). At 3 h after *S. frugiperda* infestation, the expression of *PAL4* reached a peak, and increased by 7.70-fold for ZD958 and 8.14-fold for JG218, relative to the control leaves. *CHS6* (*Zm00001d016014*) also showed a similar tendency as *PAL4* (**Figure 9E**). After *S. frugiperda* infestation, *CHS6* had a significant difference between the *S. frugiperda*-infested and the control leaves from 3 h onwards for ZD958 (F = 744.339, df =7, *P* = 0.002), while a significant difference between the *S. frugiperda*-infested and the control leaves at 3 and 6 h for JG218 (F = 487.082, df =7, *P* = 0.001), and with a peak level of 3.87-fold for ZD958 and 4.41-fold for JG218 at 6 h, respectively. LOXs play an important role in the biosynthesis of JA (**Figure 9B**). After *S. frugiperda* infestation, the expression level of *LOX1* (*Zm00001d042541*) also had a significant difference between the *S. frugiperda*-infested and the control leaves from 3 h onwards for both maize cultivars and increased by 64.13-fold for ZD958 (F = 2292.333, df =7, *P* = 0.003) and 83.79-fold for JG218 at 3 h (F = 2601.748, df =7, *P* = 0.000), relative to the control leaves (**Figure 9E**). In the ABA biosynthetic pathway, NCED is the key enzyme (**Figure 9C**). After *S. frugiperda* infestation, the expression level of *NCED9* (*Zm00001d013689*) first decreased at 3 h, then increased from 6 to 12 h for both maize cultivars and had a significant difference between the *S. frugiperda*-infested and the control leaves at 12 h for ZD958 (F = 35.94, df =7, *P* = 0.001), while a significant difference between the *S. frugiperda*-infested and the control leaves at 3 and 12 h for JG218 (F = 35.705, df =7, *P* = 0.001) (**Figure 9E**). BXs are associated with DIMBOA biosynthesis (**Figure 9D**). The expression level of *BX12* (*Zm00001d029353*) continued to increase from 0 to 12 h for both maize cultivars after *S. frugiperda* infestation and had a significant difference between the *S. frugiperda*-infested and the control from 3 h onwards (**Figure 9E**). At 12 h, the levels of *BX12* in the *S. frugiperda*-infested leaves of ZD958 (F = 624.264, df =7, *P* = 0.002) and JG218 (F = 303.731, df =7, *P* = 0.000) were 8.09 and 8.95 times as high as the control, respectively.

## 4 Discussion

### 4.1 Rapid generation of excessive MDA and H_2_O_2_ in maize leaves’ response to *S. frugiperda* infestation

Plants show various responses to stress, including morphological, biochemical, and molecular traits. Under stressful conditions, it is often found that excessive hydrogen peroxide production and oxidative pressure related with high H_2_O_2_ concentrations and antioxidant mechanisms that help scavenge redundant reactive oxygen species (ROS) in plants (Choudhury et al., 2013). The primary symptom of plants to biotic stress is the activation of their defensive systems, such as ROS generation, especially H_2_O_2_. In plant cells, when H_2_O_2_ enters the cell membrane, destructive processes are started and biological molecules are oxidized, consequently resulting in cell apoptosis (Quan et al., 2008). Herbivore attack often increases ROS generation in plants, including H_2_O_2_ (Tyagi et al., 2022). Greater accumulation of MDA indicates enhanced production of ROS. Our results showed that, after *S. frugiperda* infestation, both maize cultivars accumulated more H_2_O_2_ compared with their corresponding control plants from 0 to 6 h; compared with the control, the MDA content was significantly higher at 3 h for ZD958, while significantly higher at 3 and 6 h for JG218. As already mentioned, the higher concentration of H_2_O_2_ probably result in peroxidative cell membrane damage. It is consistent with the increased MDA content. Furthermore, after *S. frugiperda* infestation, the maximum increase of H_2_O_2_ and MDA contents was greater in JG218 (33.88% for H_2_O_2_ and 51.09% for MDA) than that in ZD958 (21.00% for H_2_O_2_ and 27.32% for MDA). It suggested that the oxidative damage of cell membrane in JG218 was more serious than that in ZD958, and JG218 was more susceptible to *S. frugiperda* than ZD958. It is evidenced that the larval weight gain ratio of JG218 leaves (0–83.28%) was higher than that of ZD958 leaves (0–70.64%) within 24 h.

### 4.2 SOD, POD, and CAT participate in maize plant’s defensive response to *S. frugiperda* infestation

It is well-known that antioxidant mechanisms are the effective ROS scavenging systems. SOD, POD, and CAT are three antioxidant enzymes that play a crucial role in maintaining a healthy balance between the production and elimination of ROS (Nadarajah, 2020). SOD catalyzes the dismutation reaction of superoxide into H_2_O_2_, and POD and CAT act on the peroxide to reduce it. In contrast, CAT increases resistance to the cell wall and triggers the expression of defensive genes (Zhao et al., 2016). Induction of antioxidative enzymes in plants by herbivory has attracted more and more attentions (War et al., 2012). In cowpea plants, the higher SOD and POD levels were observed in *S. frugiperda* injured leaves of the landrace Juti which showed higher resistance levels (Costa et al., 2020). In cucurbits, after melon fly infestation, in healthy and infected tissues of chayote and bottle gourd (resistant fruits) compared with bitter gourd, snake gourd, and cucumber (susceptible cucurbits fruits), higher activities of SOD, POD, polyphenol oxidase (PPO) and CAT were observed (Somegowda et al., 2021). After gall wasp *Dryocosmus kuriphilus* (GWDK) infestation, at all three stages of development, the activities of the POD and SOD enzymes were higher in the resistant chestnut variety leaves than they were in the susceptible chestnut variety leaves. On the other hand, the activities of the CAT and PPO enzymes were mostly higher in the susceptible chestnut variety leaves (Zhu et al., 2022). In present study, both the *S. frugiperda*-infested and the control leaves, from 0 h onwards, the activities of SOD, POD, and CAT in ZD958 were significantly higher than that in JG218 at the same point of time, except for CAT at 6 h in the *S. frugiperda*-infested leaves, which is compatible with the above mentioned that the activities of antioxidant enzymes are higher in resistant ones. Furthermore, SOD activities in both maize cultivars tended to significantly increase and then decrease to the level of the control, POD activities in ZD958 also tended to significantly increase and then decrease to the level of the control while in JG218 tended to increase and then decrease but remained significantly higher compared with the control, and CAT activities were significantly decreased and then increased to the level of the control. It suggested that maize plants activated their antioxidant enzyme system after *S. frugiperda* infestation and the damage to maize plants was stabilized at the late time. The results were further evidenced by the increase and subsequent decrease of lipid peroxidation product MDA accompanied by H_2_O_2_ contents in the *S. frugiperda*-infested leaves with the time prolong.

### 4.3 Phenolic and flavonoid compounds involved in maize plant’s defensive response to *S. frugiperda* infestation

Apart from antioxidant enzymes, plants also have a mechanism for synthesizing defensive substances in response to invading herbivores, such as plant secondary metabolites. Plant secondary metabolites are essential for plant–environment interactions. They are produced synthetically and can also be induced by biotic and abiotic stresses. Of the secondary metabolites, polyphenols are the most widely distributed and significant class of plant stress-resistant compounds (Singh et al., 2021). Additionally, polyphenols are crucial in the cyclic reduction of ROS, which, in turn, trigger a series of reactions that initiate the activation of defense-related enzymes (Blokhina et al., 2003; Nadarajah, 2020). According to chemical structure, phenolic acids, flavonoids, stilbenes, and lignans are the major groups of polyphenols which have been actively engaged in protecting plants against herbivores (Divekar et al., 2022). Many research have already reported that the accumulation of phenolics and flavonoids in plants makes them resistant to a variety of abiotic and biotic stresses. Specifically, cucurbits with melon fly infestation tissues, compared with healthy and apparently healthy tissues, had significantly higher total phenolic and flavonoid levels (Somegowda et al., 2021). Phenolic acids and flavonoids, particularly quercetin, have also increased in white cabbage upon infestation by cabbage butterflies and flea beetles (Kovalikova et al., 2019). These reports are line with our results that the total phenolic and total flavonoid contents were higher in the *S. frugiperda*-infested leaves compared with the control leaves, except for the total flavonoid contents at 6 h. It indicated that maize plants initiated a defensive response after *S. frugiperda* infestation. Furthermore, after *S. frugiperda* infestation, the changes of total phenolic and total flavonoid contents were greater in JG218 than those in ZD958, suggesting that ZD958 had higher resistance than JG218. In addition, POD is responsible for the catalytic synthesis of lignins and oxidative phenols, which contribute to the strengthening of the cell structure and make it challenging for insects to insert their mouth stylets (de Lima Toledo et al., 2021). In this study, after *S. frugiperda* infestation, the increase of POD activities and total phenolic contents is in agreement with that of previous reports. Moreover, after *S. frugiperda* infestation, the level of puncture force increased in both maize cultivars also indicated that the plant cell structure was strengthened.

### 4.4 DIMBOA is an important defensive compound in maize plant’s defensive response to *S. frugiperda* infestation

The members of the most extensively studied class of defensive compounds, benzoxazinoids, are crucial to plant resistance to numerous pathogens and pests (Wouters et al., 2016). They are found in the plant family Poaceae (formerly Gramineae) and some dicots, mostly during the early stages of plant development. The main benzoxazinoid found in young maize plants is 2,4-dihydroxy-7-methoxy-1,4-benzoxazin-3-one (DIMBOA), which is considered to be the most important chemical factor in resistance to leaf-feeding insects (Santiago et al., 2017; Kudjordjie et al., 2019). 2-(2,4-Dihydroxy-7-methoxy-1,4-benzoxazin-3-one)-β-d-glucopyranose (DIMBOA-Glc) as well as 2-hydroxy-4,7-dimethoxy-1,4-benzoxazin-3-one glucoside (HDMBOA-Glc) and 6-methoxy-benzoxazolin-2-one (MBOA) were the products of benzoxazinoid biosynthesis (Shavit et al., 2018). DIMBOA is released when plant tissues are damaged either by humans or by nature. Therefore, pathogen infections, insect feeding, and artificial plant damage may cause DIMBOA production (Zhou et al., 2018). In durum wheat, after 96 h of infestation, both *Sitobion avenae* and *Rhopalosiphum padi* aphid feeding significantly induced the predominant metabolites DIMBOA and its glycosylated form DIMBOA-Glc (Shavit et al., 2018). In maize leaves, at 24 h, HDMBOA-Glc and MBOA had the highest induction levels and were significantly upregulated. DIMBOA and DIMBOA-Glc were significantly reduced with continued *O. furnacalis* feeding (Guo et al., 2017; Guo et al., 2019). In this study, significant increases were observed for DIMBOA contents in the *S. frugiperda*-infested leaves and then followed by a significant decline at a later time, and the DIMBOA contents in ZD958 were significantly higher than those in JG218 at the same point of time, except at 6 h in the *S. frugiperda*-infested leaves. It suggested that ZD958 had higher resistance than JG218. The DIMBOA contents first significantly increased and then significantly decreased, which could be explained in such a way that *S. frugiperda* induced the accumulation of DIMBOA first, and then DIMBOA turned into DIMBOA derivatives.

### 4.5 JA might play a central role in *S. frugiperda*-induced signaling

Not only do phytohormones play an important role in plant development but also they serve as defense against various herbivores (Erb et al., 2012; War et al., 2012; Ku et al., 2018). The phytohormones JA and SA play a significant role in plant defense through multiple signal transductions (Ding and Ding, 2020; Ghorbel et al., 2021; Wang et al., 2021). JA is regarded as a key player in the induction of plant responses against herbivore attacks (Koo and Howe, 2009; Bosch et al., 2014; Escobar-Bravo et al., 2019) through the induction of a variety of defensive substances like polyamines, quinones, terpenoids, alkaloids, phenylpropanoids, glucosinolates, and antioxidants (Zhou and Memelink, 2016; Eisenring et al., 2018). Similarly, by a variety of metabolic processes, SA also regulates plant growth and defense (War et al., 2012)—for instance, to reduce the damage caused by the cotton bollworm *Helicoverpa armigera*, the SA signaling pathway in tomato produces various antioxidant enzymes (Peng et al., 2004). It is evident that SA induces more resistance against insects that penetrate and suck, whereas JA is primarily involved in defense against herbivores that eat leaves by chewing (Schweiger et al., 2014; Aljbory and Chen, 2018). The cross-talk between the ABA signaling pathway and other phytohormones like cytokinin, JA, and SA can alter how plants interact with herbivorous insects (Guo et al., 2016)—for instance, both wheat and rice plants were attacked by hessian fly, which resulted in an increase of SA, 12-oxo-phytodienoic acid, and JA but a decrease of ABA (Zhu et al., 2011). The content of JA, SA, and ABA in potato was significantly increased under aphid stress, suggesting that the cross-talks of phytohormones defend against aphid attack (Quandahor et al., 2020). In pepper plants, after an aphid attack, JA and its metabolite JA-Ile increased significantly, the SA levels remained unaltered until 7 days post-infestation, and the ABA content tended to rise later in the aphid infestation (Florencio-Ortiz et al., 2020). In soybean plants, despite the fact that aphid infestation resulted in higher SA levels in all soybean genotypes, the constitutive elevated levels of JA and ABA were an important factor in soybean tolerance to soybean aphids (Chapman et al., 2018). These results suggested that the phytohormones typically interact antagonistically or synergistically, acting in concert. In this study, *S. frugiperda* is a polyphagous chewing pest. The JA content of the infested leaves was significantly increased in JG218 from 3 h onwards while in ZD958 from 6 h onwards after *S. frugiperda* infestation, which was up to 2.52 and 1.53 times as high as the control. The SA content of both maize cultivars first significantly decreased at 3 h and then increased to the level of the control at a later time. The ABA content did not appear to be different between the *S. frugiperda*-infested and control leaves of ZD958 while significantly lower at 3 h and significantly higher at 12 h in the infested leaves of JG218, indicating that JA had a central role in *S. frugiperda*-induced signaling with overlapping and synergistic effects on SA and ABA pathways. Our results agree that the JA pathway is typically initiated by chewing insects, and JA signaling is the fundamental pathway that controls the plants’ defensive responses to herbivores (Schweiger et al., 2014; Howe et al., 2018). Previous studies have shown that the biosynthesis of alkaloids, terpenoids, and phenylpropanoids, among other secondary metabolites, is thought to be related to JA in plants (Bali et al., 2019). Furthermore, it has been reported that JA is able to induce the activities of antioxidant enzymes such as SOD, POD, CAT, and ascorbate peroxidase in plants under stresses (Sirhindi et al., 2016; Zhu et al., 2022). The increase in total phenolic and total flavonoid contents and SOD and POD activities in this study suggest that JA might play a vital role in inducing plant responses against *S. frugiperda* attack through the induction of various defensive compounds and antioxidant enzymes.

### 4.6 Genes associated with *PAL4*, *CHS6*, *BX12*, *LOX1*, and *NCED9* play an important role in maize plant’s defensive response to *S. frugiperda*

Understanding the resistance mechanisms and identifying potent genes involved in plant defense mechanisms rely heavily on gene expression and post-transcriptional regulation (Zhang et al., 2019). Genomic reprogramming takes place as a result of the infestation, which ultimately controls the synthesis of proteins or the production of secondary compounds. It has been demonstrated that benzoxazine plays a significant role in maize’s pest resistance. DIMBOA is the main benzoxazinoid found in maize. *BX* genes are associated with DIBOA biosynthesis pathway (Tzin et al., 2017). In this study, *BX12* catalyzing the reaction of DIMBOA-Glc to HDMBOA-Glc was highly upregulated in the *S. frugiperda*-infested leaves, which is consistent with previous results, such that four genes associated with benzoxazinone synthesis were highly elicited when armyworms attacked maize (Yang et al., 2020). During *Ostrinia furnacalis* feeding in maize, all *BX* genes, with the exception of *BX1*, *BX5*, *BX7*, and *BX8*, were significantly upregulated (Guo et al., 2019). These results indicate that the most prevalent responsive defense compound in maize plants is benzoxazine. In the plant defensive pathway, the phenylpropanoid pathway occupies a central position (Yadav et al., 2020). PAL is the initial enzyme in the phenylpropanoid pathway (Dehghan et al., 2014) and also responds to the phytohormones methyl jasmonate, ethylene, JA, and SA. Another significant enzyme in the phenylpropanoid cascade is CHS, and it catalyzes flavonoid biosynthesis (Dehghan et al., 2014). Phenylpropanoids have previously been shown to be induced to accumulate in the tissues of cucurbits in response to melon fly infestation, white cabbage exposed to cabbage butterflies, and flea beetles and cotton fed with *Helicoverpa armigera* and *Spodoptera litura* (Kovalikova et al., 2019; Dixit et al., 2020; Somegowda et al., 2021). In this study, on certain time points after *S. frugiperda* infestation, *PAL4* and *CHS6* were significantly upregulated, and the accumulation of total phenolics and total flavonoids was significantly increased. It has a similarity with a previous finding that suggests that the transcript levels of the genes in the phenylpropanoid pathway and the phenolic acid, proanthocyanidin, and tannin accumulation in cotton rise after an infestation of chewing insects (Dixit et al., 2020). Moreover, PAL also plays a role in the biosynthesis of SA (Lefevere et al., 2020). A study in tomato found that *PAL* gene associated with SA pathway was significantly upregulated at each time interval after exposure to aphids (Hanan et al., 2020). However, compared with the control, the content of SA in both maize cultivars only significantly decreased at 3 h after *S. frugiperda* infestation; no significant difference was observed at the other time points in this study. The predominant function of JA in mediating plant defense against attack by chewing insects is well known, and LOX genes are involved in the upstream reaction of the JA pathway (War et al., 2012). In this study, *LOX1* gene involved in the biosynthesis of JA was significantly induced in both maize cultivars by *S. frugiperda* infestation. This is in line with previous studies which found that most genes, including *LOX1*, involved in the JA pathway in maize were significantly upregulated following *O. furnacalis* herbivory (Guo et al., 2019). Two JA-related genes *LOX* and *OPR3* were significantly induced in bean plants fed by pea leafminer larvae for 4 days (Yang et al., 2021). The hormone ABA is important in regulating the relations of plant and abiotic stress. 9-*cis*-epoxycarotenoid dioxygenase (NCED) plays a key role in the ABA biosynthesis pathway (Han et al., 2004). The role of ABA in plant–herbivore interactions has gained attention—for example, upon infestation by striped stem borer, the *β-carotene hydroxylase* and *NCED* genes were significantly upregulated in rice seedlings at 3 or 6 h (Li et al., 2020). Additionally, *NCED* gene expression significantly increased following *O. furnacalis* feeding (Guo et al., 2019). In our study, *NCED9* was significantly upregulated in both maize cultivars only at 12 h after *S. frugiperda* infestation. This suggests that ABA has been a part of maize’s defensive response since the beginning of the *S. frugiperda* infestation. Combined with the total phenolic, flavonoid, and DIMBOA contents, the JA, SA, and ABA levels, and the *PAL4*, *CHS6*, *BX12*, *LOX1*, and *NCED9* expression levels, we found that whether or not the accumulation of these substances synchronized with the expression level of these genes depended on the upstream, midstream, or downstream genes involved in the biosynthetic pathway that they regulate. The relationship of JA, SA, and ABA is an interesting topic to be further explored.

## 5 Conclusion

In conclusion, our results demonstrated that *S. frugiperda* rapidly triggered off the antioxidative response of maize plants, and JG218 was more sensitive to *S. frugiperda* than ZD958. It is mainly reflected by the following aspects: first, the H_2_O_2_ and MDA contents of infested leaves were rapidly and significantly increased and then decreased to the level of the control. Compared with the control leaves, the puncture force values and the total phenolic, total flavonoid, and DIMBOA contents of infested leaves were significantly increased as well within a certain time. The SOD and POD activities of infested leaves were significantly increased in a certain period of time, while the CAT activities decreased significantly and then increased to the control level. The JA levels of infested leaves were significantly improved, whereas the SA and ABA levels changed less. Genes associated with phytohormones and defensive substances including *PAL4*, *CHS6*, *BX12*, *LOX1*, and *NCED9* were significantly induced at certain time points, especially *LOX1* whose function needs to be further studied. Most of these parameters changed greater in JG218 than in ZD958. Moreover, the larvae bioassay showed that *S. frugiperda* larvae weighed more on JG218 leaves than those on ZD958 leaves. It can make us understand better the resistance mechanisms of maize plants to *S. frugiperda*, providing a theoretical basis for developing crop cultivars’ defense against herbivores and for pest management for sustainable crop production.

## Data availability statement

The original contributions presented in the study are included in the article/**Supplementary Material**. Further inquiries can be directed to the corresponding authors.

## Author contributions

XJ and HL contributed to the conception of the study and project administration. JY, CM and RJ conducted the experiments and data analyses. HL and JY wrote the manuscript. XJ revised the manuscript. HY, YZ and HZ gave advice. All authors carefully read, revised, and approved the submitted version.
