# Supplementary material for: Different responses of two maize cultivars to Spodoptera frugiperda (Lepidoptera: Noctuidae) larvae infestation provide insights into their differences in resistance
Source: Front Plant Sci. 2023 Feb 10;14:1065891. doi: 10.3389/fpls.2023.1065891 (PMC9950569; doi:10.3389/fpls.2023.1065891)
Supplement: Supplementary file 1 [file Table_1.docx]

Supplementary Material

# Supplementary Tables

| **File 1 Gene-specific primers for qRT-PCR** | |
| --- | --- |
| **Gene name** | **Primer sequence (5’-3’)** |
| *Zm00001d051166* | Forward: GGCTTGCCCTCCAACCTGTC |
|  | Reverse: GTCCTGGTTGTGCTGCTCCG |
| *Zm00001d029353* | Forward: CAACAACCTTGGCTCCCT |
|  | Reverse: GAAACATCTCGCCGCACT |
| *Zm00001d042541* | Forward: TTCCAAACAGCATCTCCATT |
|  | Reverse: GCCTTATTACAACAGTCCTCACG |
| *Zm00001d013689* | Forward: CTTCCCGTCCCCATCTCA |
|  | Reverse: GCTGCCACTATCCCAATTTTAC |
| *Zm00001d016014* | Forward: AGAACGCTGACGGGGAGA |
|  | Reverse: GGCAATAGTGTGGCAGATAAAC |
| *Zm00001d010159* | Forward: TATCCAGGCTGTTCTTTCGTT |
|  | Reverse: CATTAGGTGGTCGGTGAGGT |
